# The complete chloroplast genome of *Libocedrus chevalieri*, a Critically Endangered species in New Caledonia

**DOI:** 10.1080/23802359.2021.1964399

**Published:** 2021-08-13

**Authors:** Jiaxin Pang, Wanlong Hu, Wentao Wang, Jialiang Li, Kangshan Mao

**Affiliations:** Key Laboratory of Bio-Resource and Eco-Environment of Ministry of Education, College of Life Sciences, State Key Laboratory of Hydraulics and Mountain River Engineering, Sichuan University, Chengdu, People’s Republic of China

**Keywords:** Complete chloroplast genome, *Libocedrus chevalieri*, phylogenetic analysis, Cupressaceae

## Abstract

*Libocedrus chevalieri* is a rare endemic conifer from New Caledonia, and it is evaluated as Critically Endangered in the IUCN Red List of Threatened Species. To take conservation actions more effectively, a survey of its genomic background and evolutionary status is of great significance. Illumina paired-end reads were used to character the chloroplast (cp) genome of *L. chevalieri*. The circular genome is 122,068 bp in length, containing 115 genes, in which include 83 protein-coding genes, four ribosomal RNA genes, and 28 transfer RNA genes. Nine genes (*atp*F, *rpo*C1, *ndh*B, *ndh*A, *rpl*2, *pet*B, *rpl*16, *pet*D, *rps*12) have one intron, whilst one gene (*ycf*3) has two introns. Inverted repeat (IR) sequence doesn’t exist in the genome. The GC content of the cp genome is 34.1%. The phylogenetic analysis demonstrates that *L. chevalieri* has a close relationship with *L. plumosa*.

*Libocedrus chevalieri* is a rare conifer in Cupressaceae, and has been classified as Critically Endangered in the IUCN Red List of Threatened Species (Thomas 2010). The species is a shrub or small tree to 5 m tall and grows in high-altitude maquis shrubland at altitudes of 1,450 to 1,600 m (Farjon 2010; Eckenwalder 2009; Thomas et al. 2018). *L. chevalieri* only occur in New Caledonia on Grande Terre where is regarded as one of the world’s 25 biodiversity hotspots (Myers et al. 2000). However, none is known about the species’ genomic background. In this study, we used Illumina paired-end sequencing data to assemble the complete cp genome of *L. chevalieri*, and surveyed its phylogenetic status.

Dried leaf sample were obtained from the Royal Botanic Garden Edinburgh Herbarium (http://data.rbge.org.uk/herb/E00215054, Markus Ruhsam, MRuhsam@rbge.org.uk, voucher number: E00215054), which were collected from New Caledonia (Latitude: 21°52′49″S, Longitude: 166°25′21″E). The whole-genome re-sequencing was conducted on the Illumina Hiseq Platform (Illumina, San Diego, CA, USA), which was deputed to BGI (https://www.genomics.cn/). After removing the adapters and low-quality reads, 3902 Mb high-quality clean reads were yielded.

GetOrganelle (Jin et al. 2020) was used to assemble the cp genome of *L. chevalieri* with the published cp genome of *L. plumosa* (Sudianto et al. 2020) as a reference, and PGA (Qu et al. 2019) was used to annotate the circular genome with the default parameters. Geneious Prime 2021.1.1 (https://www.geneious.com) was used to visualize the cp gene and correct the annotation result. The complete cp genome sequence was uploaded in GenBank under accession number MZ169380.

The whole cp genome of *L. chevalieri* is 122,068 bp in length, containing 115 genes, in which include 83 protein-coding genes, four ribosomal RNA genes, and 28 transfer RNA genes. Nine genes (*atp*F, *rpo*C1, *ndh*B, *ndh*A, *rpl*2, *pet*B, *rpl*16, *pet*D, *rps*12) have one intron, whilst one gene (*ycf*3) has two introns. Inverted repeat (IR) sequence doesn’t exist in the genome. The GC content of the cp genome is 34.1%.

To reveal the phylogenetic status of *L. chevalieri* in Cupressaceae, we downloaded nine cp genome data of species belonging to Cupressaceae from National Center for Biotechnology Information (NCBI, https://www.ncbi.nlm.nih.gov/). The sequences were aligned by MAFFT ver. 7 (https://mafft.cbrc.jp/alignment/server/index.html), and a maximum likelihood (ML) tree was reconstructed via MEGAX (Kumar et al. 2018) with 1000 bootstrap replicates. The ML tree (Figure 1) suggest that *L. chevalieri* is sister to *L. plumosa* and this relationship received a bootstrap support as high as 99.

**Figure 1.**
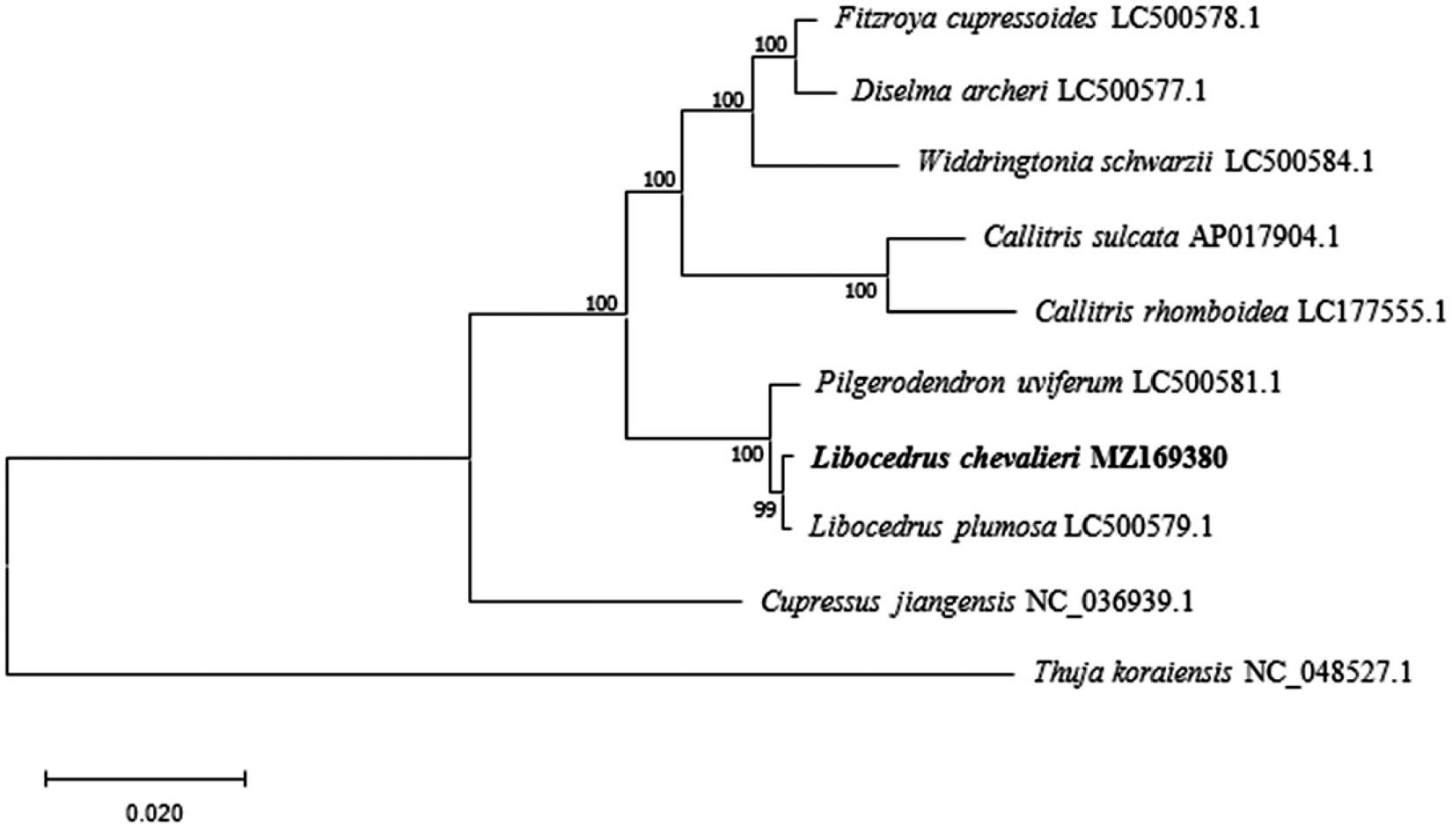
Phylogenetic relationships of *Cupressaceae* using nine chloroplast genome sequences based on the maximum likelihood (ML) analysis. GenBank accession numbers: *Cupressus jiangensis* (NC_036939.1), *Thuja koraiensis* (NC_048527.1), *Libocedrus plumosa* (LC500579.1), *Pilgerodendron uviferum* (LC500581.1), *Widdringtonia schwarzii* (LC500584.1), *Diselma archeri* (LC500577.1), *Fitzroya cupressoides* (LC500578.1), *Callitris sulcata* (AP017904.1), *Callitris rhomboidea* (LC177555.1).

In conclusion, the complete cp genome of this Critically Endangered species, *L. chevalieri*, not only provides genomic background support for its future conservation actions, but also contributes to the further phylogenetic and evolutionary studies of *Libocedrus*, Cupressaceae and conifers.

## Data Availability

The genome sequence data that support the findings of this study are openly available in GenBank of NCBI (https://www.ncbi.nlm.nih.gov/) under the accession number MZ169380. The associated BioProject, SRA, and Bio-Sample numbers are PRJNA741855, SRX11271992 and SAMN19910176, respectively.
